# Bead-based immunoassay allows sub-picogram detection of histidine-rich protein 2 from *Plasmodium falciparum* and estimates reliability of malaria rapid diagnostic tests

**DOI:** 10.1371/journal.pone.0172139

**Published:** 2017-02-13

**Authors:** Eric Rogier, Mateusz Plucinski, Naomi Lucchi, Kimberly Mace, Michelle Chang, Jean Frantz Lemoine, Baltazar Candrinho, James Colborn, Rafael Dimbu, Filomeno Fortes, Venkatachalam Udhayakumar, John Barnwell

**Affiliations:** 1 The Centers for Disease Control and Prevention, Center for Global Health, Division of Parasitic Diseases and Malaria, Malaria Branch, Atlanta, GA, United States of America; 2 Programme National de Contrôle de la Malaria, Ministère de la Santé Publique et de la Population (MSPP), Port-au-Prince, Haiti; 3 National Malaria Control Program, Maputo, Mozambique; 4 Clinton Health Access Initiative, Boston, Massachusetts, United States of America; 5 National Malaria Control Program, Luanda, Angola; National Institutes of Health, UNITED STATES

## Abstract

Detection of histidine-rich protein 2 (HRP2) from the malaria parasite *Plasmodium falciparum* provides evidence for active or recent infection, and is utilized for both diagnostic and surveillance purposes, but current laboratory immunoassays for HRP2 are hindered by low sensitivities and high costs. Here we present a new HRP2 immunoassay based on antigen capture through a bead-based system capable of detecting HRP2 at sub-picogram levels. The assay is highly specific and cost-effective, allowing fast processing and screening of large numbers of samples. We utilized the assay to assess results of HRP2-based rapid diagnostic tests (RDTs) in different *P*. *falciparum* transmission settings, generating estimates for true performance in the field. Through this method of external validation, HRP2 RDTs were found to perform well in the high-endemic areas of Mozambique and Angola with 86.4% and 73.9% of persons with HRP2 in their blood testing positive by RDTs, respectively, and false-positive rates of 4.3% and 0.5%. However, in the low-endemic setting of Haiti, only 14.5% of persons found to be HRP2 positive by the bead assay were RDT positive. Additionally, 62.5% of Haitians showing a positive RDT test had no detectable HRP2 by the bead assay, likely indicating that these were false positive tests. In addition to RDT validation, HRP2 biomass was assessed for the populations in these different settings, and may provide an additional metric by which to estimate *P*. *falciparum* transmission intensity and measure the impact of interventions.

## Introduction

Detection of *Plasmodium* sp. proteins has been utilized for malaria diagnostic purposes since the 1990s, and has greatly aided in malaria treatment and elimination through the field-deployable point-of-care RDT (or immunochromatographic test, ICT)[1]. Rapid diagnostic tests are continuing to supplant microscopy as the most common malaria diagnostic test worldwide[2], and have allowed for significant progress in attaining the goal of universal confirmation of all suspect malaria cases. The most successful of these tests identifies the presence of the *P*. *falciparum*-specific protein HRP2, which is abundantly produced at multiple stages during its humanerythrocytic cycle, accumulates in the erythrocyte cytoplasm, and is released into plasma during erythrocyte rupture [3, 4]. The HRP2 protein was first identified in the avian malaria parasite *P*. *lophurae*[5], and later found to be produced by only one of the human malarias[6, 7], making it a valuable marker for falciparum identification[8, 9]. However, quickly following the development of the field-deployable test, it was found that some persons cleared of parasitemia were still capable of testing positive to HRP2 up to a month following successful treatment, leading to reduced confidence in the test as a genuine or reliable diagnostic [10–13]. In the absence of a confirmatory diagnostic test, such as microscopy or PCR, the true positivity of the RDT test for active falciparum infection cannot be ascertained as HRP2 persists after infection has cleared.

In addition to its role in clinical malaria diagnosis, presence of HRP2 in a population can also provide an estimate of *P*. *falciparum* transmission intensity, as those with circulating protein provide an indication of active or very recent infection [14]. This metric could prove valuable in regions of the world with a high percentage of all malaria infections being low-density and having asymptomatic parasite carriage. Currently, the standard immunoassay for HRP2 detection is the sandwich ELISA which has been found to reliably have a detection limit of around 1ng/mL [15, 16]. Using this same concept of antigen capture and detection by monoclonal antibodies, we developed an inexpensive bead-based assay for HRP2 which utilizes the flow cytometry based Luminex® platform. In direct comparison with the HRP2 ELISA, we found sensitivity of the bead-based assay to be increased over 200-fold when assaying for purified rHRP2 and HRP2 produced by *P*. *falciparum* cultured strains. The bead assay allowed for biosamples to be assayed with minimal dilution, and provided excellent specificity in detecting only HRP2-producing malaria infections over a range of different parasitemias. In evaluating the performance of field-collected HRP2 RDT results, ultrasensitive HRP2 detection by the bead assay provided insight into the true performance of the tests in places of different *P*. *falciparum* transmission intensities, and was able to distinguish the HRP2 biomass among different age groups in these settings. The addition of a highly-sensitive assay for HRP2 to the researcher’s toolbox will provide external validation of RDT tests being used in the field, and further malaria surveillance efforts with a powerful and reliable tool to estimate *P*. *falciparum* circulation in a human population.

## Materials and methods

For all human samples, participant consent was written.

### Binding of capture antibody to polystyrene beads

Monoclonal IgM (MPFM-55A, Abcam; ab9206) antibodies were covalently bound to polystyrene BioPlex® COOH beads (BioRad; 1715060XX) by the commonly-used EDC/Sulfo-NHS intermediate reaction. Reactive esters were formed on the carboxylated beads in the presence of EDAC [1-Ethyl-3-(3ʹ-dimethylaminopropyl)carbodiimide](EMD Millipore; 341006) and Sulfo-NHS [N-hydroxysulfosuccinimide] (ThermoScientific; 24510) under light agitation for 20 min. Carboxyl to antibody amine crosslinking took place in the presence of activation buffer (LuminexCorp; 11–25171) under light agitation for 2 h. Nonspecific protein binding was blocked by BSA incubation (PBS pH7.2, 0.05% Tween20 [PBS-T] + 1% BSA) for 30 min and beads resuspended in blocking buffer with the addition of 0.02% NaN_3_. Once the optimal coupling concentration of capture antibody to beads was found (S1 Fig), a large-scale bead coupling was done to cover all experiments.

### Capture and detection assay

**Fig 1:**
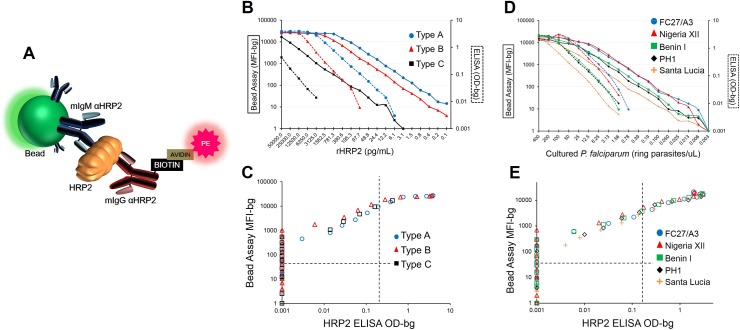
Comparison of bead-based HRP2 assay with HRP2 ELISA. (**A**) Completed scaffolding of bead complex when HRP2 is present in a sample. (**B**) Serial dilutions of Type A, B, and C rHRP2 assayed by bead assay (solid lines) and ELISA (hashed lines), and (**C**) comparison of signal intensities of same dilutions between bead assay and ELISA. (**D**) Serial dilutions of cultured *P*. *falciparum* strains assayed by bead assay (solid lines) and ELISA (hashed lines), and (**E**) scatterplot for signal intensities of same dilutions between immunoassays. Vertical and horizontal dashed lines in (**C**) and (**E**) represent lower limits of reliable detection for the ELISA and bead assay, respectively.

If HRP2 was present in a sample, a bead complex would be formed as illustrated in Fig 1A. Samples to be assayed were diluted in blocking buffer containing 0.5% Polyvinyl alcohol (Sigma; P8136), 0.8% Polyvinylpyrrolidine (Sigma; PVP360), 0.1% casein (ThermoFisher; 37528), 0.5% BSA (Sigma; A9418), 0.3% Tween-20, 0.05% sodium azide, and 0.01% *E*. *coli* extract to prevent non-specific binding. Before assaying, diluted samples were centrifuged to pellet debris. Reagent diluent consisted of PBS-T plus 0.5% BSA, 0.02% sodium azide. Filter bottom plates (Millipore; MABVN1250) were pre-wetted with PBS-T and approximately 1,500 coupled beads incubated with sample for 1.5 h under gentle shaking. Wells were washed (3x between all incubations), and incubated for 45 min with biotinylated detection antibody (mouse IgG anti-HRP2, MPFG-55P, Abcam; ab9206)(antibody previously biotinylated by ThermoScientific EZ-Link Micro Sulfo-NHS-Biotinylation Kit according to manufacturer’s protocol; 21925). Wells subsequently incubated with streptavidin-phycoerythrin (Invitrogen; S866) for 30 min. Wells had a final wash incubation with reagent diluent for 30 min, resuspended in 100 uL PBS, and were read on a Bio-Plex 200 machine (BioRad; 171000201) by generating the median fluorescence signal for 50 beads and then the mean fluorescence intensity (MFI) of the medians among replicates. The final measure, denoted as MFI-bg, was reported by subtracting MFI values from beads on each plate only exposed to sample diluent during the sample incubation step. Testing the assay under different reagent concentrations allowed for the determination of the optimum conditions for assay sensitivity (S1 Fig).

### ELISA assay

The same dilutions prepared for the bead assay were also assayed for HRP2 by ELISA for direct comparison. The CelLabs Malaria Antigen (HRP2) CELISA (CelLabs, New South Wales, Australia) was employed as the comparison immunoassay, and assays were run according to manufacturer’s protocol. Briefly, samples were added for 1 h, wells washed and incubated with conjugate antibody (1:200x) for 1 h and washed again. Chromogen substrate was added, color allowed to develop for 15 min, and reaction stopped by addition of Stopping Solution and read on spectrophotometer (Molecular Devices SpectraMAX 340PC, Sunnyvale, CA, USA) at 450 nm. Signal intensity for sample is indicated as optical density (OD) for sample well minus OD of blank wells (OD-bg). As recommended by the manufacturer, the OD value at which a sample is considered positive is 0.2 OD units above the negative control provided in the ELISA kit. As the negative control consistently provided low OD values, we considered any OD-bg value above 0.2 to be ‘HRP2 positive’ by this ELISA assay.

### Sample collection

Where indicated, participant consent was written. Samples from U.S. resident blood donors were used to represent a population of persons known to not have been to a malaria-endemic area in the past 6 months and, thus, not exposed to *P*. *falciparum*. All blood samples were from consenting adults who had screened negative for HIV and hepatitis B viruses and had no reported history of international travel in the last 6 months.

Blood samples from travelers returning to the U.S. after travelling to malaria endemic areas are submitted to CDC Malaria Branch for reference diagnostic support and surveillance purposes. Plasma obtained from these samples was used after identifying information was removed. To determine *Plasmodium* species and parasitemia of these samples, qPCR was performed utilizing the Rougemont method as previously described [17]. Use of samples obtained in U.S. were determined to be non-engagement in human subjects research as approved by CDC Human Subject Office.

Previously-collected, anonymized samples from two cross-sectional household surveys in holoendemic Nampula Province, Mozambique were tested on the novel assay platform. Dried blood spots on Whatman 903 filter paper and RDTs (SD Bioline Malaria Ag Pf, Standard Diagnostics, 05FK50) were collected from all consenting household members in a representative sample of households in Mecubúri and Nacala-a-Velha Districts in 2013 and 2014 [18, 19]. Persons found to be RDT positive were treated with anti-malarials according to study protocol. The Mozambique protocol was reviewed and approved by the country’s National Bioethics Review Board.

Dried blood spot samples from therapeutic efficacy surveys in Angola in 2013 and 2015 were collected on Whatman 903 filter paper of assenting children prior the course of antimalarial treatment from Zaire, Benguela, Uige, and Lunda Sul Provinces with microscopically-confirmed *P*. *falciparum* infections. The HRP2 concentrations from pre-treatment samples were compared with the parasite density as measured by microscopy (2015) and qPCR (2013) [20]. Samples from a 2016 Angola Health Facility Survey were gathered by teams which visited 90 randomly selected public health facilities in Huambo and Uíge Provinces in Angola. Exit interviews were performed on randomly selected patients attending the outpatient department in each health facility. Patients were tested with malaria RDTs (SD Bioline Pf/Pv, 05FK80) by survey teams, and blood from consenting patients was collected on Whatman 903 filter paper. Angolan surveys were determined to be non-research by the Angolan Ministry of Health (MOH) and the CDC Human Subject Office.

Haitian samples were collected in early 2015 from a nationwide tracking results continuously (TRaC) nationwide household-based cluster survey. Upon consent or assent, all persons participating in the survey were assigned a 6-digit identification number that could not be traced back to the individual. Blood spots were collected on Whatman 903 filter paper and RDTs preformed (First Response Ag *P*. *falciparum* (HRP2) Card Test, Premier Medical Corporation Ltd., I12FRC25XX). Persons found to be RDT positive were treated with anti-malarials according to protocol approved by the Haitian MOH and CDC Human Subject Office.

As quality of biomolecules is known to degrade with poor storage conditions, careful considerations were taken in the collection and storage of DBS from the Mozambique, Angola, and Haiti surveys. For all three surveys, the Whatman 903 cards were dried for a minimum of 2 hours and individually packaged in air-tight plastic baggies with individual desiccants. Samples were stored in dry conditions in a temperature controlled room until processing at CDC labs.

### Recombinant HRP2 proteins

Three recombinant HRP2 (rHRP2) proteins were used in attempt to quantify limit of detection for the bead assay. Amino acid sequences for the HRP2 protein (recombinant or native) are classified as Type A, B, or C depending upon the product of the Type 2 and Type 7 epitope repeats [21]. The Type A rHRP2 (Thio-S-FCQ79-HRP2) was provided by the Army Malaria Institute (Brisbane, Australia), Type B (GST-W2-HRP2) provided by MicroMol GmbH (Karlsruhe, Germany), and Type C by CTK Biotech (San Diego, CA, USA).

In order to standardize quantification of recombinant HRP2 protein from different sources, determination of rHRP2 was achieved by optical density A280 readings, and solving for concentration by the Beer-Lambert law:

$$A_{i280}=\varepsilon_{i}{\text{c}}_{i}l$$

                                Where: *A*_*i*280_ = absorbance reading at 280 nm

                                              ℇ_*i*_ = molar attenuation (extinction) coefficient for protein (mL/g∙cm)

                                              *c*_                                *i*_ = molar concentration of protein (g/mL)

                                              l = length of cuvette (cm)

Samples of rHRP2 were diluted in PBS and read on spectrophotometer at wavelength of 280 nm. The final calculated concentration for the rHRP2 sample was an average of three readings. Polyacrylamide gels of rHRP2 samples shown in S2 Fig.

### *P*. *falciparum* reference strain culture

*P*. *falciparum* reference strains were cultured in T-75 flasks at 10%HCT in CRPMI 10% O+ serum, gas and incubate in 37° incubator, with parasitemias of blood cultures determined daily via blood smears. Blood cultures were harvested when parasitemias of culture flasks were above 1.0%, with content of culture flasks collected to a 50 ml centrifuge tube, spun down, and supernatant removed. Pellets were washed with RPMI1640 twice, and after the last wash, supernatants removed and the pellets re-suspended to 50% hematocrit with RPMI 1640. Of the suspension, 500uL was dispensed into a 1.5ml tube for calculation of protein concentration with two tubes of these prepared. All suspensions were then stored in -80°C until use. After protein concentration is determined, aliquots of each strain for the panel were prepared accordingly, diluting each *P*. *falciparum* suspension with human whole O+ blood.

### Statistical analysis

To determine the relationship between parasite density and HRP2 concentration, data were fit to a linear regression model as shown in S3 Fig by the SAS PROC GLM command. Comparisons of the novel bead assay to the RDT test involved the formations of ROC curves through the SAS PROC LOGISTIC command with the ROC statement.

## Results

### Direct comparison with HRP2 ELISA

Immobilizing antibodies on polystyrene beads allows for the capture of the HRP2 protein, and detection is then accomplished through binding with a biotinylated secondary antibody and further incubation with streptavidin-phycoerythrin (PE). If HRP2 is present in a sample, a bead complex is formed (as illustrated in Fig 1A), and PE fluorescence signal is proportional to the amount of HRP2 captured. For the analyses presented here, these fluorescence values are all displayed as median fluorescence intensity minus background fluorescence (MFI-bg). Previous studies utilizing the HRP2 ELISA have found wide variations in the limit of detection for the assay ranging from 0.11 to 3.9 ng/mL[15, 16, 22, 23]. As protein detection through immunoassays relies on antibody/antigen binding, we tested the three different classified forms of recombinant HRP2 (rHRP2): Type A, B, and C. Type A rHRP2 has the highest quantity of known epitopes for monoclonal antibodies, whereas Type B has fewer, and Type C fewer still (S1 Table). Screening rHRP2 by the bead assay was found to give detection limits unique for each rHRP2—Type A: 0.24 pg/mL, Type B: 1.43 pg/mL, Type C: 71.9 pg/mL. As 50uL of sample was tested in each well for the assay, this represents an absolute mass of 12, 72, and 3,595fg of protein, respectively. These limits of detection suggests a performance advantage over the HRP2 ELISA with greater titrating capacity and overall limit of detection by a factor of 200–400x (Fig 1B and 1C), depending on rHRP2 Type. Testing of native HRP2 produced by cultured *P*. *falciparum* reference strains provided similar findings, with the bead assay showing a dramatically-improved detection capacity when titrating out the HRP2 protein, and allowing a 100–300x lower limit of detection in direct comparison with the ELISA assay (Fig 1D and 1E).

### Specificity of bead HRP2 assay and correlation with parasitemia

**Fig 2:**
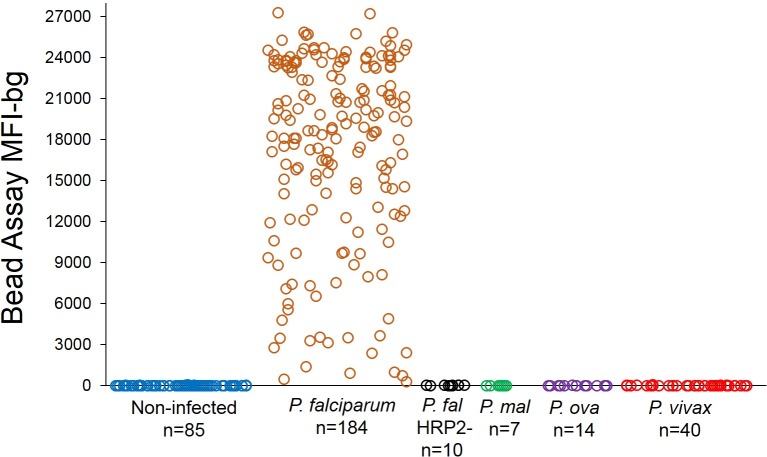
HRP2 bead assay only detects HRP2-producing *P*. *falciparum*. Plasma from non-infected persons, or persons with symptomatic *Plasmodium* spp. infection was assayed at a 1:10x dilution for presence of HRP2. **P. mal**: *P*. *malariae*; **P. ova**: *P*. *ovale*.

The HRP2 bead assay was shown to be exceptionally specific, only providing substantial fluorescence signal if the HRP2 protein was present in the sample (Fig 2). Isolates of *P*. *falciparum* lacking a functional HRP2 gene (an increasing global concern [24, 25]), isolates from non-falciparum *Plasmodium* sp. infections, and samples from non-infected US residents showed negligible fluorescence signal by the assay. An additional advantage of the flow cytometry-based Luminex® platform was observed to be the dynamic range of fluorescence detection, with a scale of 1 to 28,000 fluorescence intensity (MFI) units for the HRP2 assay. For persons presenting with symptomatic *P*. *falciparum* infection, the majority of plasma samples gave a MFI-bg signal at the upper end of the machine’s detection capacity (range: 150–27,277 MFI-bg units; mean: 17,378; s.d.: 6,928).

A significant positive correlation was observed between P. falciparum infection densities and HRP2 concentration (S3 Fig), as has been reported previously [26, 27], but with variation regardless of whether parasitemia was determined through light microscopy (R^2^ = 0.285, p<0.001) or nucleic acid-based qPCR methods (R^2^ = 0.091 and 0.193, p<0.001). Estimates for the additional amount of HRP2 per additional parasite ranged from 2.1 to 5.6 femtograms, which was similar to the previously-published estimate of 5.2 fg released from a single *P*. *falciparum* parasite over the course of an erythrocytic replication cycle[22]. The overall variation in HRP2 concentrations for infected persons is not surprising, as individuals would have been infected with varying parasite densities and for different lengths of time, allowing different production and accumulation levels of the protein. Also, different *P*. *falciparum* strains are known to produce different protein variants, and quantities, of the HRP2 protein [28–30] (see Fig 1D).

### Cutoff determination for appropriate positive signal by bead assay

As with many laboratory assays, the HRP2 bead assay is subjected to a degree of background signal and needed to be determined at which signal threshold determines true positivity for the presence of HRP2 protein. Following a sample’s subtraction of the fluorescence signal for the bead/capture antibody complex (MFI-bg), rHRP2 and culture supernatant titrations all continued to a fluorescence signal of zero (Fig 1B and 1D), showing a total absence of non-specific binding and signal. As blood samples were measured throughout this study, the intention was to find a threshold signal above which the researcher could confidently state the presence of HRP2 protein. Threshold determination was attempted by assaying a large panel of known blood samples where the individual had no opportunity for *P*. *falciparum* infection. Eighty-five US resident blood donor samples were assayed at a 1:10x dilution of whole blood, and the histogram of fluorescent signal shown in S4 Fig with attempted fittings of parametric distributions by SAS v9.3 PROC UNIVATIAVE command with HISTOGRAM statement with statistical appropriateness of a Type I error (α) threshold of 0.01. The lognormal, gamma, and gumbel distributions were found to be statistically appropriate for this population of US residents. As the mean MFI-bg signal for the sample population was below zero, the cutoff signal for HRP2 positivity was determined to be five standard deviations of the lognormal distribution added to zero, giving a MFI-bg value of 36. This method makes no assumptions about the signal distribution for persons actually carrying the HRP2 protein, only that the positive signal for the bead assay would be well outside of the distribution of known negatives. Though simple, this method gives high statistical confidence in correctly identifying a sample’s signal as indicative of HRP2 presence. Extensive community sampling from malaria-endemic nations of Haiti and Mozambique gave similar mean and standard deviation estimates of the MFI-bg signal for the probable HRP2-negative populations within those samplings (S5 Fig).

### The HRP2 bead assay for determining field-based RDT accuracy

Rapid diagnostic test results are sometimes compared against microscopy or PCR tests in an attempt to evaluate their performance for detection of active infection [14, 31–33]. Though the point-of-care diagnostic capacity of RDTs is certainly the most practical among these three tests, comparison among platforms should involve appropriate interpretation, as all three are essentially inspecting for different biological components of a parasite. As the HRP2-based RDT is simply a chromatographic immunoassay for the antigen, comparison with an additional HRP2 test of improved sensitivity could provide assessment of how well the RDT is truly preforming in the field. To this end, we examined samples collected from three areas of varying *P*. *falciparum* endemicity to ascertain the concordance between these two immunoassays for the HRP2 protein and estimate the limits of detection for the separate HRP2-based RDTs in the different settings.

**Fig 3:**
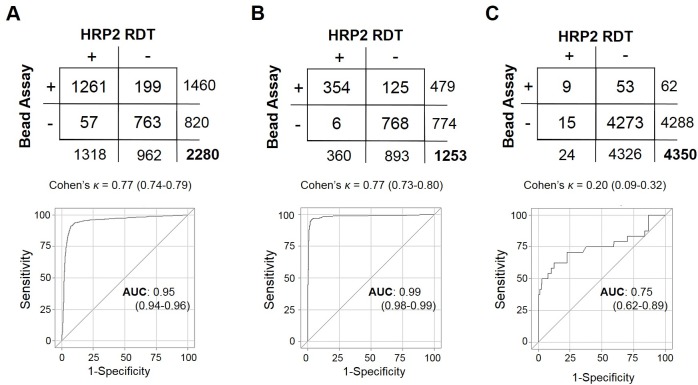
Test comparison between bead assay and HRP2 RDTs. (**A**) Persons from Mozambique tested for presence of HRP2 by RDT (SD Bioline Pf) and bead assay, and comparison statistics for the two tests. For this purpose, the RDT was treated as the standard classification predictor and the bead assay as the novel test. The same analysis in (**B**) for persons from Angola where the SD Bioline Malaria Ag P.f/P.v RDT was used and in (**C**) Haiti where the First Response HRP2 RDT was used.

Testing and DBS sampling of 2,280 persons from a *P*. *falciparum* holoendemic area in northern Mozambique showed 57.8% positive by HRP2 RDT and 64.0% positive by the HRP2 bead assay (Fig 3A). Overall concordance between the two tests was good, showing a kappa coefficient of 0.77 (95% CI: 0.74–0.79), but a significant difference was found in detection capacity, with the bead assay showing more positives (McNemar’s Χ^2^: 78.8, p<0.001) and a ROC area under the curve of 0.95 (0.94–0.96) when defining the bead assay as the novel classification predictor. This finding is consistent with a higher sensitivity of the bead assay as compared to RDT, as field-ready RDT units are manufactured and tested against a known level of cultured parasitemia[34] that is well above the detection limit of the bead assay (see Fig 1D and 1E). Of all tests performed, 57 (4.3%) RDT positive specimens from Mozambique showed no detectable HRP2 by the bead assay. In previous product testing, the RDT used in this survey (SD Bioline Malaria Ag Pf, 05FK50) was found to respond well with a panel detection score of 95 (out of 100) at 200p/uL falciparum cultured parasitemia and 0.0% false-positive rate for ‘clean negative’ blood not containing other *Plasmodium* spp. or known cross-reactive factors[34]. Though false positives did not arise during product testing with ‘clean negative’ blood in the laboratory setting, this does not take into account false positives that would potentially arise in the field from non-ideal storage conditions, operator error, data entry errors, or blood with cross-reactive components.

Testing and DBS sampling of 1,253 persons in a high-endemic region of Angola showed good concordance between the RDT test and bead assay, with a kappa coefficient of 0.77 (95% CI: 0.73–0.80)(Fig 3B). As with the Mozambique survey, detection capacity was increased with the bead assay (McNemar’s Χ^2^: 108.1, p<0.001) and a ROC area under the curve of 0.99 (0.98–0.99). The RDT test used in this study (SD Bioline Malaria Ag P.f/P.v, 05FK80) had also performed well in WHO product testing with a panel detection score of 92 and a 0.0% false-positive rate for the HRP2 band when testing ‘clean negative’ blood. The bead assay assisted in confirming the high specificity of this test, only showing 6 persons (0.48% of the sample population) with a positive HRP2 band on the RDT test, but no detectable HRP2 by the bead assay. It is important to note that samples collected in this Angola study were from a healthcare setting, increasingly the likelihood of symptomatic, high-density infections which would elicit higher HRP2 concentrations in the blood (see S3 Fig).

Testing and DBS sampling of 4,350 persons in Haiti, an area of historically low *P*. *falciparum* transmission[35], showed a much lower agreement between the bead assay and HRP2 RDT, with a kappa coefficient of 0.20 (95% CI: 0.09–0.32)(Fig 3C). For Haitian samples, detection capacity was also found to be increased by the bead assay (McNemar’s Χ^2^: 21.2, p<0.001) with a ROC area under the curve of 0.75 (0.62–0.89). Previous product testing had also found the RDT used in this survey (First Response *P*. *falciparum* HRP2, I13FRC25) to perform well, showing a panel detection score of 95 at parasitemia of 200p/uL and 0.4% false-positive rate when testing ‘clean negative’ blood[34]. Assuming 0.4% of the 4,350 RDTs performed during the Haiti survey were predicted to be false positives, the expectation value would be 17 false positive tests. We found 15 persons who tested positive by RDT, but showed no detectable HRP2 through the bead assay. A goodness-of-fit test found the discrepancy between expected false positives and observed RDT test positives with no HRP2 was non-significant (Χ^2^: 0.43, p = 0.49), giving evidence for the false positives being due to inherent RDT error and not operator error.

### Distribution of HRP2 detection in different *P*. *falciparum*-endemic settings

**Fig 4:**
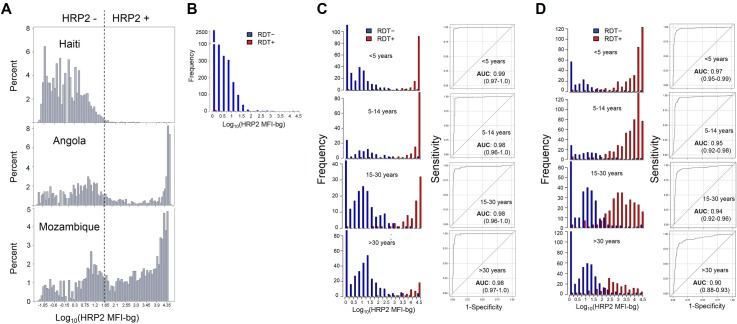
Distribution of HRP2 bead assay signal intensity by RDT result in different populations. (**A**) MFI-bg signal at log scale for entire Haiti, Angola, and Mozambique study populations. In order to show entire range of signal distribution, any sample which gave MFI-bg signal below zero had log taken of absolute value and then multiplied by -1, making negative values possible. Signal distributions by RDT test positivity given at the time of DBS sample collection for Haiti (**B**), Angola (**C**), Mozambique (**D**) surveys, with Angola and Mozambique populations providing enough RDT positives to allow for age categorization. For each age category, the bead assay was assessed at the novel classification predictor by ROC analysis and calculation of area under the curve.

In evaluating the distribution of the bead assay HRP2 signal at the population level, clear differences were seen when comparing the surveyed populations between Haiti, Angola, and Mozambique. Altogether, 1.4% of Haitians sampled were found to contain the HRP2 protein, compared to 38.2% of Angolans, and 64.0% of Mozambicans (Fig 4A). For Haitian persons, no clear separation was found in the signal intensity for those who were RDT positive versus RDT negative (Fig 4B), possibly due to the high relative percentage of likely RDT false positives (see Fig 3C). As many RDT positives were found in the Angolan population, histograms for distribution of HRP2 signal by the bead assay could be stratified into age categories, and showed clear separation of HRP2 signal between persons RDT positive and negative (Fig 4C). When using the bead assay as the novel classification predictor in comparison with the HRP2 RDT, nearly ideal classification was seen among all age categories with an ROC AUC of 0.99 or 0.98. Again, as these samples were collected in a healthcare setting, it is much more likely persons with HRP2 are symptomatic and seeking treatment, indicative of a higher-density infection. This is accentuated by the Angola histograms in Fig 4A and 4C showing high MFI-bg signal for the bead assay for most of these RDT positive persons.

In Mozambique, the distribution of signal intensity and performance of the bead assay as a classifier varied by age (Fig 4D). As these samples were collected by community-based household sampling, this provides more of an accurate representation of the HRP2 levels in the non-treatment seeking (likely asymptomatic) population. In children under 5 years of age, a clear bimodal separation of the bead assay signal for RDT negatives and positives was seen, and the bead assay provided almost ideal classification, with a ROC AUC of 0.97 (0.95–0.99). For older age categories, this separation became increasingly less pronounced, likely due to the fact older persons in holoendemic areas possess immune capacity to suppress parasitemia [36], and, thus, HRP2 accumulation (S6 Fig). Additionally, HRP2 is known to elicit a humoral response in humans [37], and it is a distinct possibility immune complexes with this antigen would lead to a lower amount of detectable HRP2 protein, as has been seen with RDT tests [38]. Though this difference bead assay signal intensity for RDT positives/negatives was the least pronounced in the oldest age category (older than 30 years), the bead assay still performed well as a classification predictor, showing a AUC of 0.90 (0.88–0.93).

## Discussion

Here we demonstrate a novel method for *P*. *falciparum* HRP2 detection that allows the protein to give a functional signal at a concentration of approximately 1 pg/mL, depending on the type of rHRP2 standard used. As this bead-based assay is formatted to a 96-well plate and uses standard immunoassay reagents, this method allows for inexpensive and high-throughput screening of samples to reliably detect the presence of HRP2. After considerations for personnel time, reagent cost, and cost of expendables, we found the bead assay to cost less than $1 USD per sample.

In direct comparison with the ELISA HRP2 assay, improved sensitivity was verified through assaying for purified rHRP2 as well as native HRP2 produced by cultured *P*. *falciparum* strains. The explanation for this substantial difference between these two immunoassays may be multifaceted, but could potentially be explained by the 3-dimensional nature of antigen capture and detection by the bead assay, or even due to the fluorescence detection system of the Luminex® platform versus ELISA optical density readings. Caution should be taken in translating HRP2 concentration of a biospecimen to a *P*. *falciparum* parasite density. Many culture-based growth studies benefit from the reliable accumulation of this protein with increased parasite mass [39, 40], but the *in vivo* accumulation and metabolism of this protein is not well elucidated and likely involves many parasite and host factors. In addition, high variability of expressed HRP2 sequences and concentrations among different *P*. *falciparum* strains [28, 41], makes standardization and predictability difficult. Nevertheless, though RDT tests are in practice an immunoassay for the HRP2 protein, these tests are validated against known cultured parasite densities, and WHO RDT product testing considers 200p/uL to be the ‘low’ value for testing against cultured parasitemia[34]. Testing *P*. *falciparum* reference strains by the bead assay provided an appreciable MFI-bg signal anywhere from 0.1 to 0.01p/uL of *in vitro* culture from these strains. However, this does not necessarily mean that a person with *in vivo* parasitemia of this range would be detected by the bead assay.

The bead assay was employed to evaluate HRP2 RDT performance in different *P*. *falciparum* transmission settings. In the holoendemic setting of northern Mozambique, high-endemic setting of Angola, and low-endemic setting of Haiti, the bead assay was found to be an exceptional classification predictor by ROC analysis. As current HRP2 RDT generally show visible bands at HRP2 concentrations exceeding 1 ng/mL[42], it was not surprising to find many persons from all surveys that were negative by RDT test, but found to have HRP2 in their blood samples by the bead assay. Though careful considerations were taken in collecting and storage of DBS during these different surveys, potential variations could exist with respect to sample integrity due to environmental conditions or length of storage until processing in the laboratory. Overall, this comparison provides an objective measure for the reliability of the HRP2-based RDT that is being employed in the healthcare or community setting as well as determining operator-related performance.

The bead assay provides a unique look into the history of *P*. *falciparum* transmission for an area due to the persistent nature of the HRP2 protein in systemic circulation [9, 11–13]. The protein’s lingering increases the window of detection beyond only when a person is actively infected. As the presence of the protein in a person is indicative of active or recent *P*. *falciparum* presence, having an estimate for the proportion of persons harboring any amount of HRP2 provides a clear depiction of what has happened in the recent past in terms of number of persons infected with this parasite. This was shown in comparing the percentage HRP2 positive persons in the Haitian and Mozambican community surveys with 1.4% of Haitians with active/recent infection versus 64.0% of Mozambicans. The enhanced sensitivity of the bead assay allows the ability to detect past infections that occurred much prior to the survey, thus expanding the window of detection. This may be most useful in very low endemic settings where residual transmission leads to persistent cycles of *P*. *falciparum*, but with high proportions of asymptomatic cases [43], and with few individuals with active infection that could be identified during a community survey.

As with all immunoassays, the quality of detection is dependent upon the antibodies and reagents available. Though providing excellent detection capacity, this assay could potentially be further improved upon by the development of novel monoclonal antibodies against HRP2. The findings of this study showed consistency among different geographical variants of the parasite, but additional studies should aim to repeat these results, as different sub-strains of *P*. *falciparum* are known to vary in their HRP2 genotype [21], and different RDT tests are feasible for different settings. Additionally, the Luminex® bead-based technology is currently not employed in many laboratories around the world, so this assay would be limited to the facilities which possess the machinery. Though these data show the application of this methodology to the malaria field, this immunoassay could also be developed for other infectious disease fields which rely on protein detection for diagnostics or surveillance.

In summary, the bead-based HRP2 assay provides the public health community with exceptional sensitivity and specificity which allows for objective testing of HRP2-based RDT reliability and performance. In addition, this lowered limit of HRP2 detection can potentially provide a useful metric in order to estimate malaria transmission in a study population.

## Supporting information

S1 Fig Optimization of reagent and sample concentration for HRP2 bead assay. In each comparison, a Blank (non-infected blood) is shown as well as plasma samples from fifteen *P*. *falciparum* infected persons. (**A**) Comparison of sample volume used during assay. (**B**) Different coupling concentrations of monoclonal IgM anti-HRP2 (clone MPFM-55A) to beads. (**C**) Different concentrations of biotinylated monoclonal IgG anti-HRP2 (clone MPFG-55P) (**D**) Different concentrations of streptavidin-phycoerythrin (Strep-PE) (**E**) Different dilutions of sample.
(TIF)

S2 Fig Recombinant HRP2 proteins used for analysis. (**A**) Polyacrylamide gel. (**B**) Sequences for Type A and Type B proteins used in study.
(TIF)

S3 Fig Correlation between *P*. *falciparum* parasitemia and circulating HRP2 concentration. (**A**) Persons from U.S. domestic surveillance with parasitemia quantified by qPCR. (**B**) Children from 2013 Angola therapeutic efficacy study (TES) with parasitemia quantified by qPCR. (**C**) Children from 2015 Angola TES with parasitemia quantified by microscopy. (**D**) Data from each data set were fitted to a linear regression model and estimates for coefficients shown. Estimates for slopes predict an increase in HRP2 concentration anywhere from 2.1 to 5.6 fg/uL for an increase of one *P*. *falciparum* parasite/uL blood at time of sample collection. Compare with previous estimate of a 5.2 fg release of HRP2 across entire lifecycle of one *P*. *falciparum* parasite [22].
(TIF)

S4 Fig Statistical modeling the distribution of MFI-bg signal from non-infected persons to determine bead assay signal appropriate to define HRP2 positivity. (**A**) Histogram of the bead assay signal from the 85 non-infected individuals in Fig 2 and overlay of various distributions fitted to the data. (**B**) Estimates of mean and standard deviation (s.d.) as provided by distributions in (**A**). Only three of the seven distributions were found to be statistically acceptable with a Type I error rate of 1%.
(TIF)

S5 Fig Statistical modeling of distribution of MFI-bg values in probable HRP2 negative populations from Haiti and Mozambique. For each dataset, samples with a MFI-bg value of >35 (positivity cutoff as determined by S4 Fig) were eliminated from analysis, and parametric distributions attempted to fit to remaining data. (**A**) Histograms for all ‘negative’ persons from Haiti and Mozambique surveys with overlay of fitted distributions (**B**) Estimates of mean and standard deviation (s.d.) as provided by distributions in (**A**). Compare with estimates generated in S4 Fig.
(TIF)

S6 Fig Loess curves for all persons from Mozambique survey as divided by a positive or negative RDT result with age maintained as continuous. Red and blue shadings represent 95% confidence intervals.
(TIF)

S1 Table Selected epitope sequences and frequency within rHRP2s used in study. (DOCX)

S2 Table Data from Recombinant HRP2s as Tested by the Bead Assay and ELISA. (XLSX)

S3 Table Data from P. falciparum Culture Strains as Tested by the Bead Assay and ELISA. (XLSX)

S4 Table Data from Bead Assay Testing Human Plasma Samples Non-infected or Infected with Plasmodium. (XLSX)

S5 Table Data from Mozambique Cross-Sectional Surveys. (XLSX)

S6 Table Data from Angola Health Facility Survey. (XLSX)

S7 Table Data from Haiti Nationwide Survey. (XLSX)
