# Supplementary figures and images for: Bead-based immunoassay allows sub-picogram detection of histidine-rich protein 2 from Plasmodium falciparum and estimates reliability of malaria rapid diagnostic tests
Source: PLoS One. 2017 Feb 13;12(2):e0172139. doi: 10.1371/journal.pone.0172139 (PMC5305216; doi:10.1371/journal.pone.0172139)

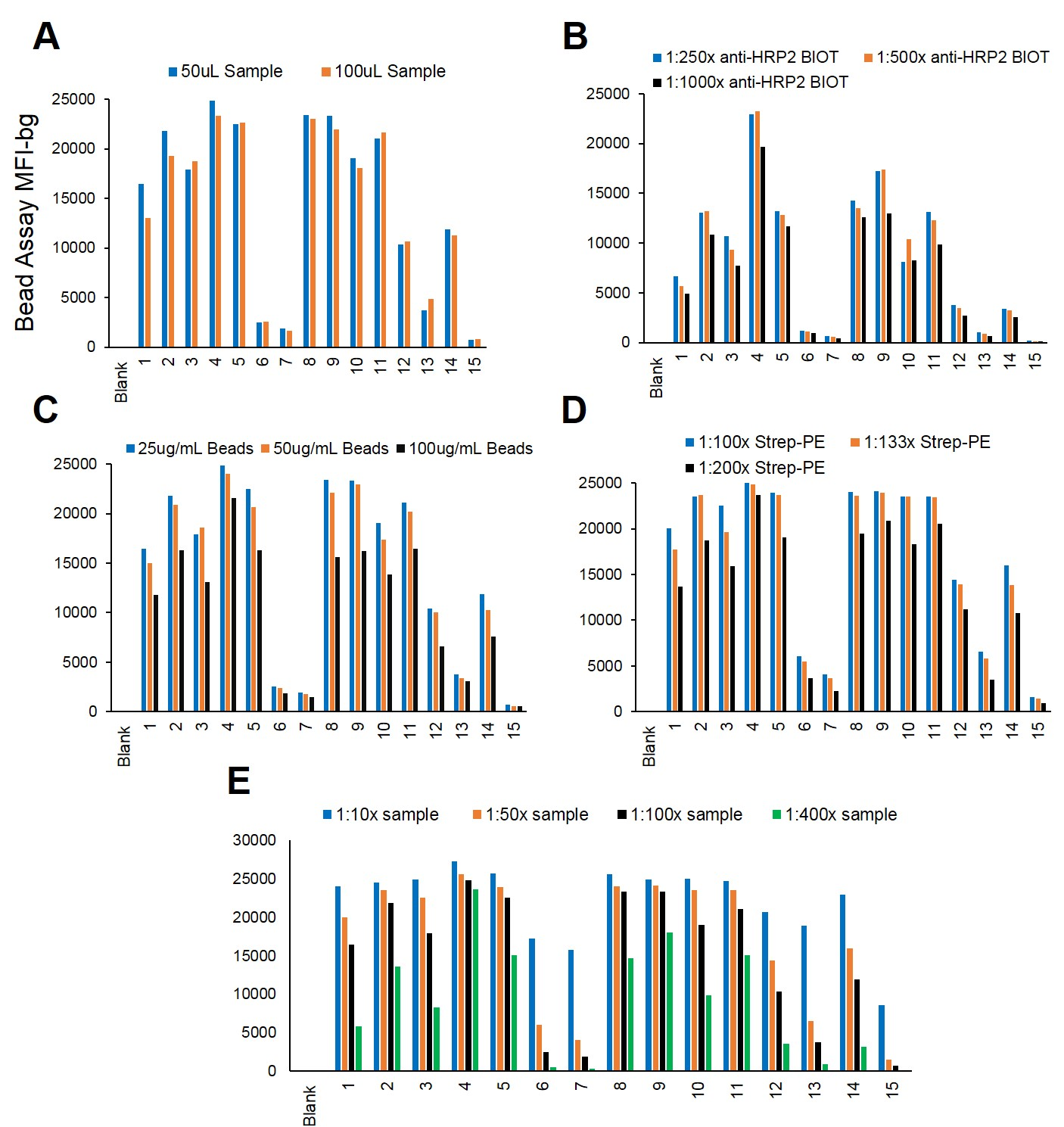

Supplement: S1 Fig — In each comparison, a Blank (non-infected blood) is shown as well as plasma samples from fifteen P. falciparum infected persons. (A) Comparison of sample volume used during assay. (B) Different coupling concentrations of monoclonal IgM anti-HRP2 (clone MPFM-55A) to beads. (C) Different concentrations of biotinylated monoclonal IgG anti-HRP2 (clone MPFG-55P) (D) Different concentrations of streptavidin-phycoerythrin (Strep-PE) (E) Different dilutions of sample. (TIF) [file pone.0172139.s001.tif]

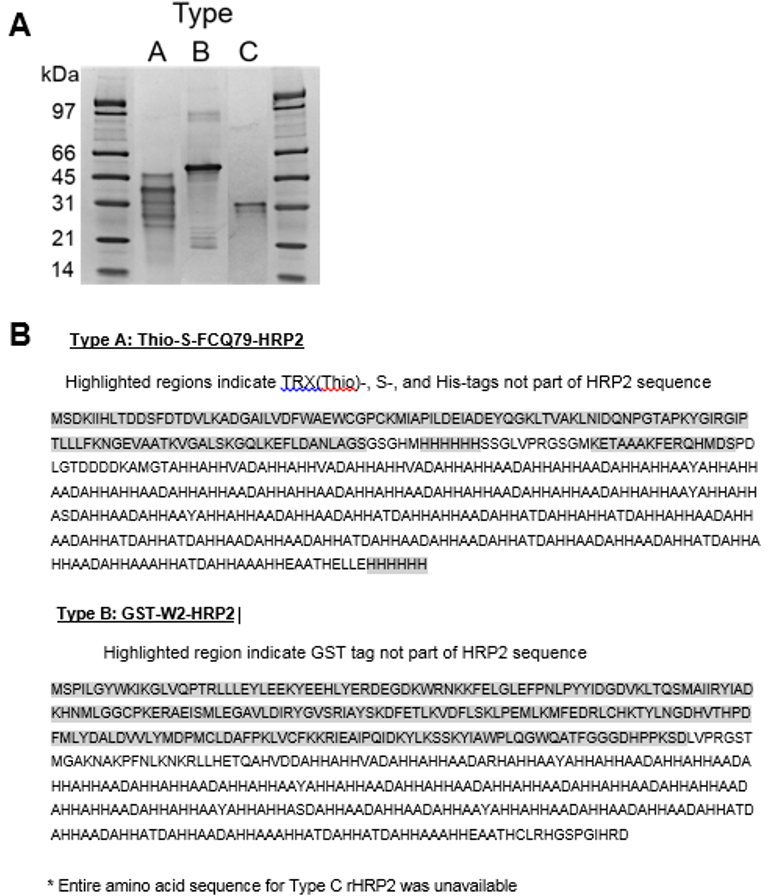

Supplement: S2 Fig — (A) Polyacrylamide gel. (B) Sequences for Type A and Type B proteins used in study. (TIF) [file pone.0172139.s002.tif]

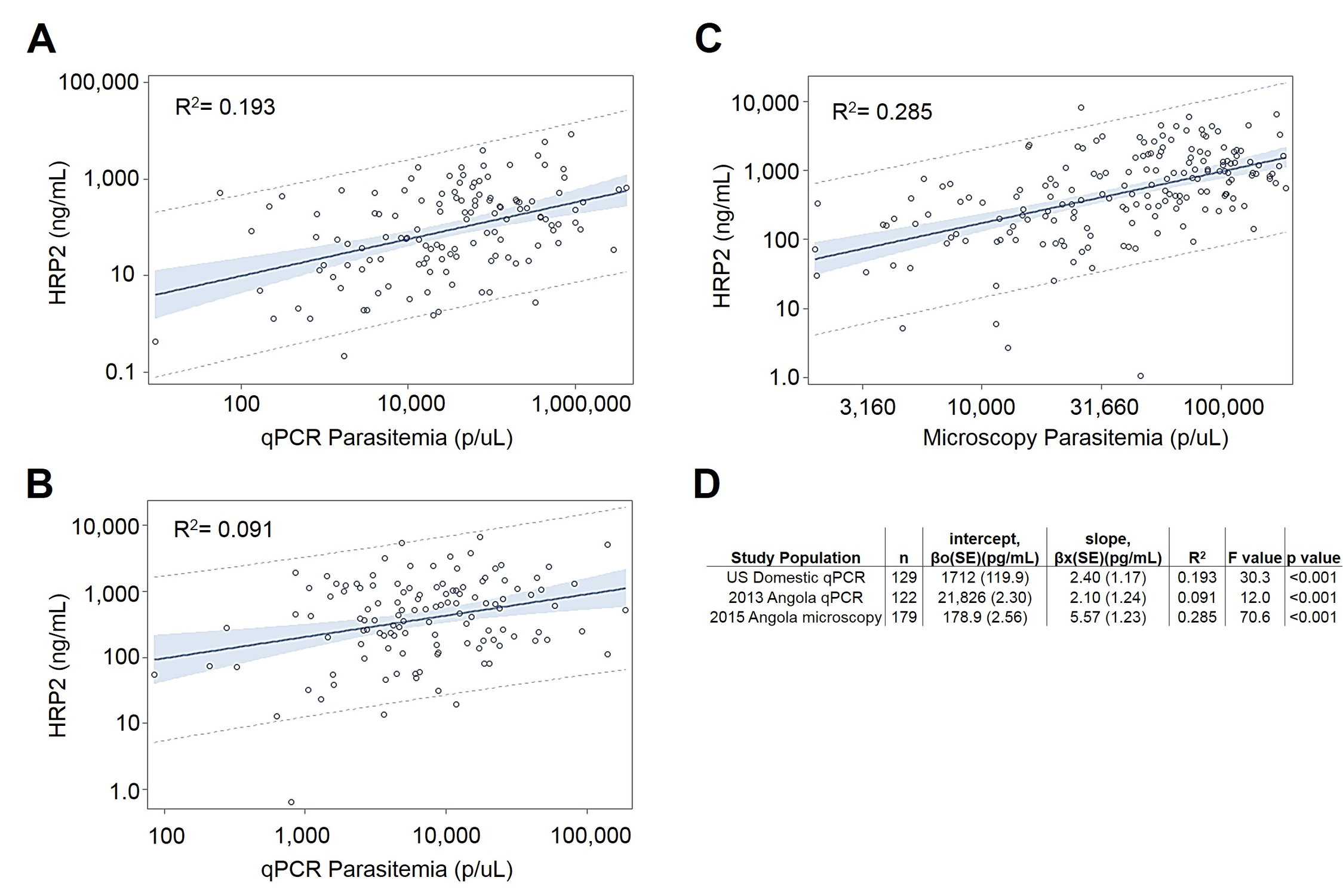

Supplement: S3 Fig — (A) Persons from U.S. domestic surveillance with parasitemia quantified by qPCR. (B) Children from 2013 Angola therapeutic efficacy study (TES) with parasitemia quantified by qPCR. (C) Children from 2015 Angola TES with parasitemia quantified by microscopy. (D) Data from each data set were fitted to a linear regression model and estimates for coefficients shown. Estimates for slopes predict an increase in HRP2 concentration anywhere from 2.1 to 5.6 fg/uL for an increase of one P. falciparum parasite/uL blood at time of sample collection. Compare with previous estimate of a 5.2 fg release of HRP2 across entire lifecycle of one P. falciparum parasite [22]. (TIF) [file pone.0172139.s003.tif]

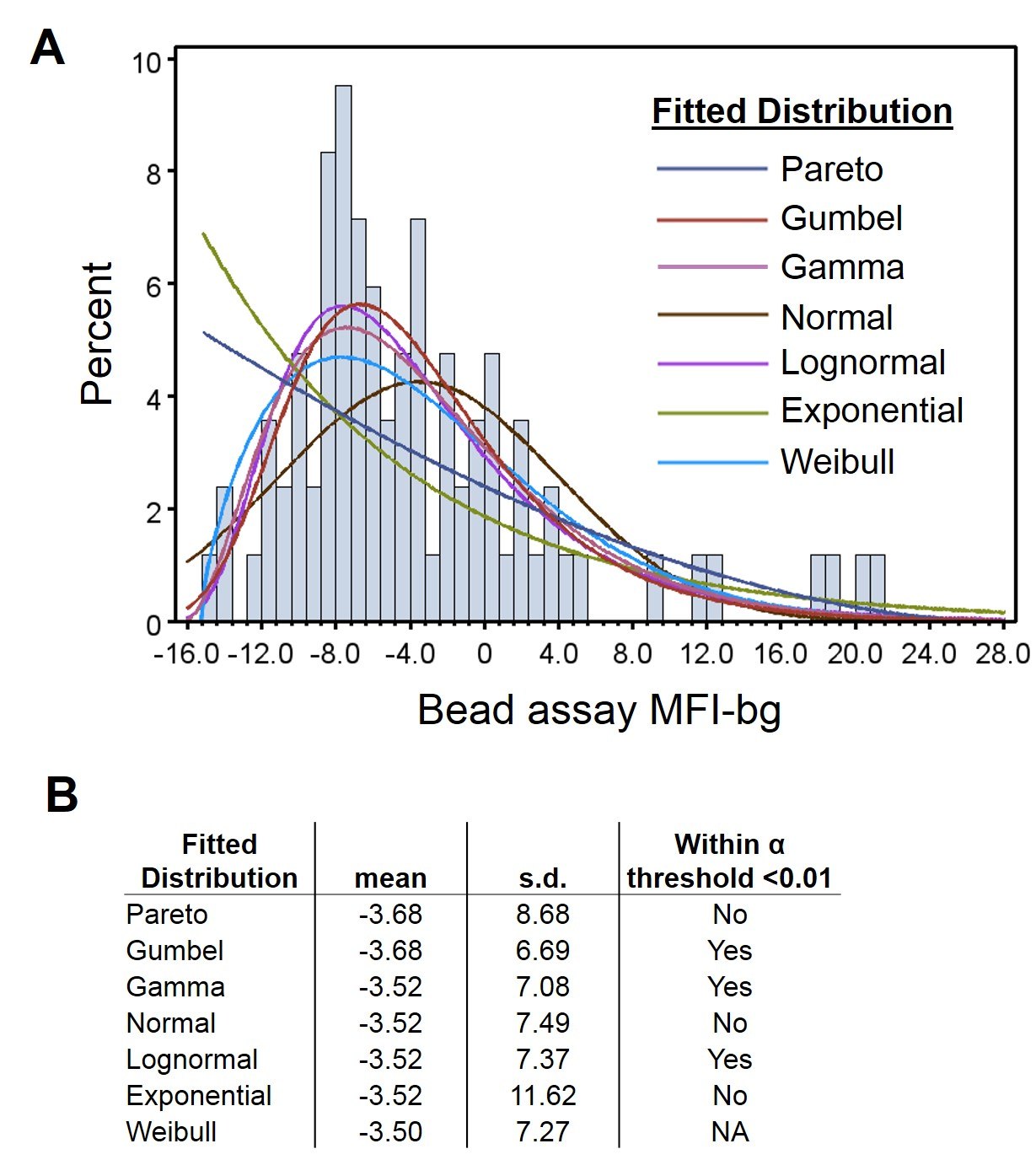

Supplement: S4 Fig — (A) Histogram of the bead assay signal from the 85 non-infected individuals in Fig 2 and overlay of various distributions fitted to the data. (B) Estimates of mean and standard deviation (s.d.) as provided by distributions in (A). Only three of the seven distributions were found to be statistically acceptable with a Type I error rate of 1%. (TIF) [file pone.0172139.s004.tif]

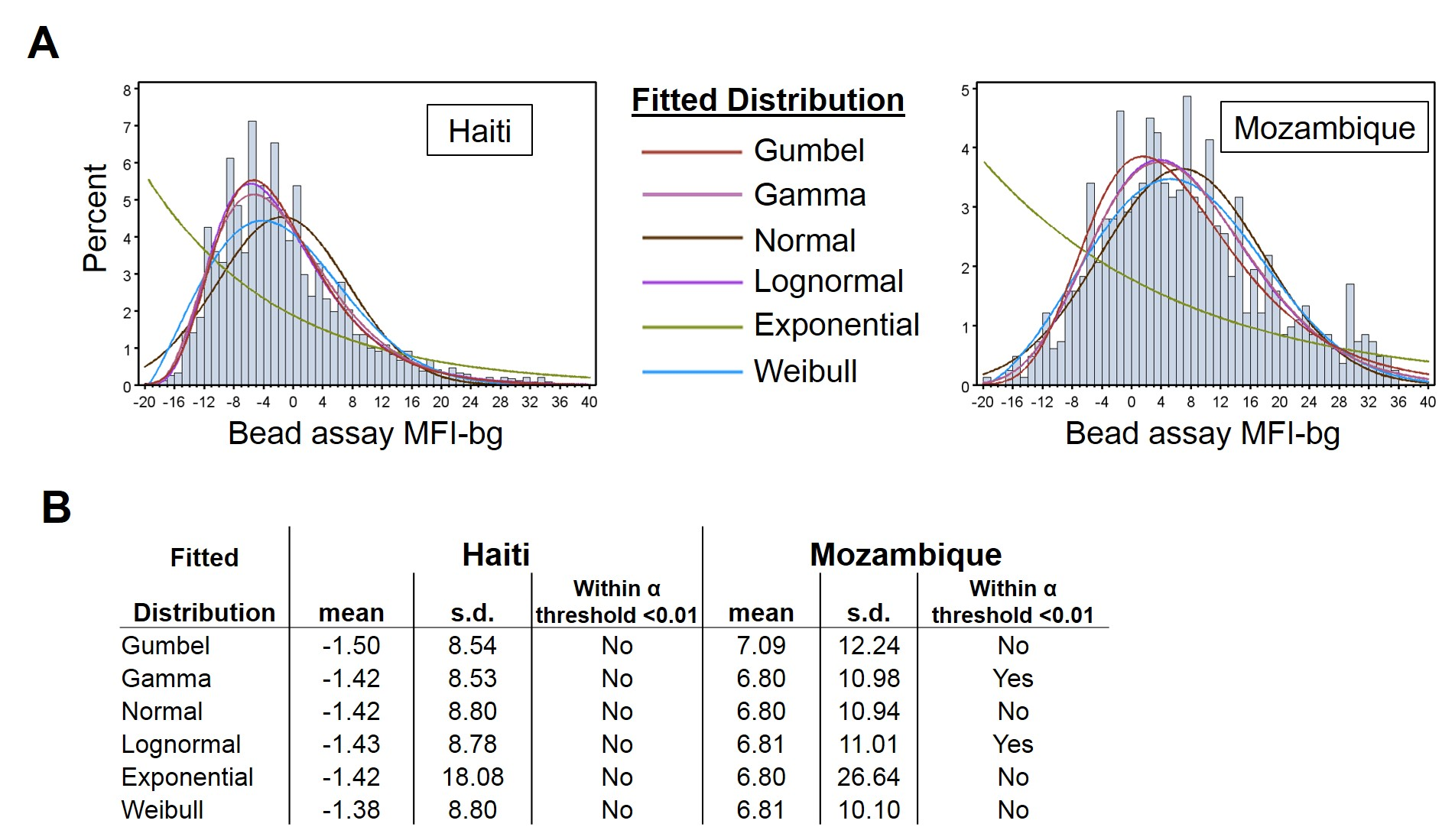

Supplement: S5 Fig — For each dataset, samples with a MFI-bg value of >35 (positivity cutoff as determined by S4 Fig) were eliminated from analysis, and parametric distributions attempted to fit to remaining data. (A) Histograms for all ‘negative’ persons from Haiti and Mozambique surveys with overlay of fitted distributions (B) Estimates of mean and standard deviation (s.d.) as provided by distributions in (A). Compare with estimates generated in S4 Fig. (TIF) [file pone.0172139.s005.tif]

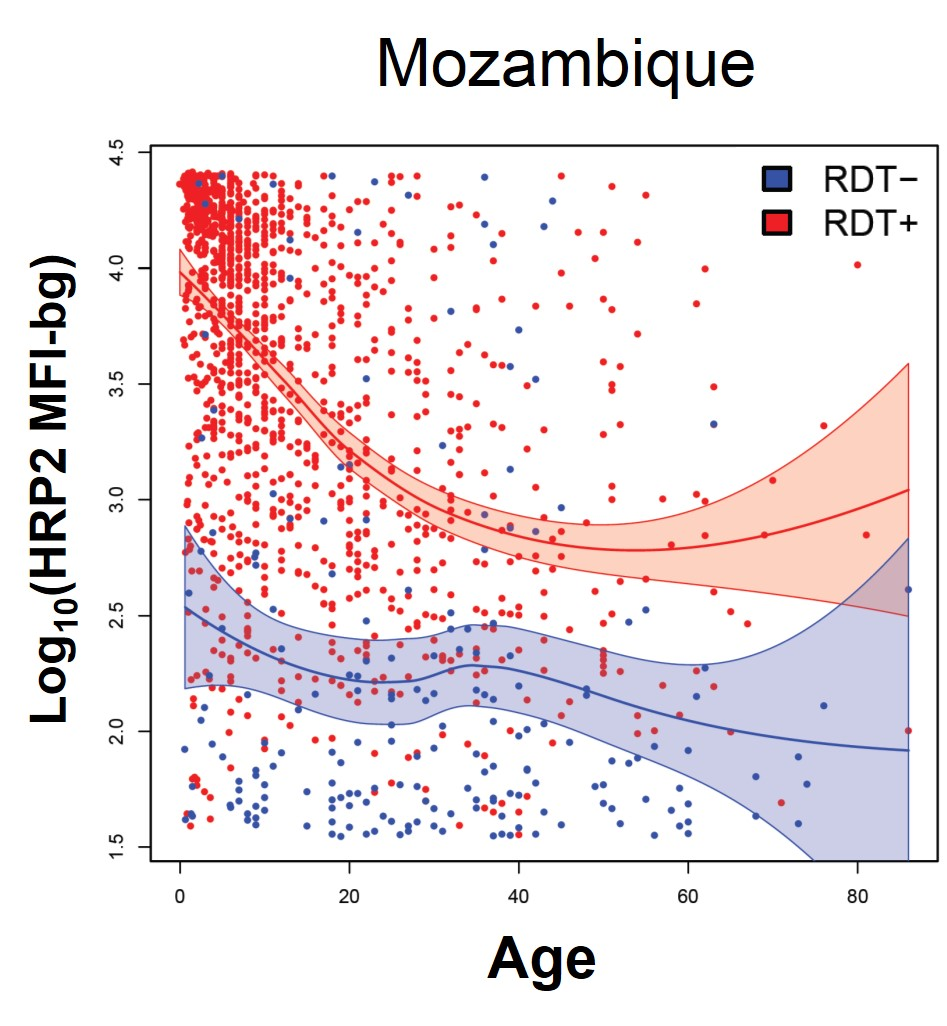

Supplement: S6 Fig — Red and blue shadings represent 95% confidence intervals. (TIF) [file pone.0172139.s006.tif]
