# Supplementary material for: Bead-based immunoassay allows sub-picogram detection of histidine-rich protein 2 from Plasmodium falciparum and estimates reliability of malaria rapid diagnostic tests
Source: PLoS One. 2017 Feb 13;12(2):e0172139. doi: 10.1371/journal.pone.0172139 (PMC5305216; doi:10.1371/journal.pone.0172139)
Supplement: S1 Table — (DOCX) [file pone.0172139.s007.docx]

**S1 Table.** Selected epitope sequences and frequency within rHRP2s used in study

| Code* | Repeat Sequence | Type A | Type B | Type C |
| --- | --- | --- | --- | --- |
| 1 | AHHAHHVAD | 3 | 1 | 3 |
| 2 | AHHAHHAAD | 13 | 12 | 10 |
| 3 | AHHAHHAAY | 2 | 2 | 1 |
| 5 | AHHAHHASD | 1 | 1 | 1 |
| 6 | AHHATD | 9 | 4 | 1 |
| 7 | AHHAAD | 9 | 6 | 3 |
| 10 | AHHAAAHHATD | 1 | 0 | 0 |
| 11 | AHN | 0 | 0 | 0 |
| 12 | AHHAAAHHEAATH | 1 | 1 | 1 |
| None | ATDAHHAHHAADAHH^†^ | 3 | 0 | unknown |
| None | **AHHAHHAADAHHAA**^†^ | 3 | 1 | unknown |

* Repeats within HRP2 protein from global isolates of *P. falciparum* [1]

^†^ Identified from previous study for the specific paring of the mouse anti-HRP2 clones

MPFG-55P/MPFM-55A for immunoassay. Bold was identified as major epitope [2]

Type 2 repeat x Type 7 repeat score:

Type A = 13 x 9 = 117

Type B = 12 x 6 = 72

Type C = 10 x 3 = 30

**References**

1. Baker J, Ho MF, Pelecanos A, et al. Global sequence variation in the histidine-rich proteins 2 and 3 of Plasmodium falciparum: implications for the performance of malaria rapid diagnostic tests. Malaria journal **2010**; 9:129.
2. Kumar N, Pande V, Bhatt RM, et al. Genetic deletion of HRP2 and HRP3 in Indian *Plasmodium falciparum* population and false negative malaria rapid diagnostic test. Acta Tropica **2013**; 125:119.
